# Caspase recruitment domain family member 10 regulates carbamoyl phosphate synthase 1 and promotes cancer growth in bladder cancer cells

**DOI:** 10.1111/jcmm.14683

**Published:** 2019-09-29

**Authors:** Xi Liu, Xiaotong Zhang, Jianbin Bi, Zhenhua Li, Zhe Zhang, Chuize Kong

**Affiliations:** ^1^ Department of Urology The First Affiliated Hospital China Medical University Shenyang China

**Keywords:** CARD10, CPS1, NF‐κB pathway, urinary bladder cancer

## Abstract

Bladder cancer, which can be divided into non‐muscle‐invasive and muscle‐invasive bladder cancer, is the most common urinary cancer in the United States. Caspase recruitment domain family member 10 (CARD10), also named CARD‐containing MAGUK protein 3 (CARMA3), is a member of the CARMA family and may activate the nuclear factor kappa B (NF‐κB) pathway. We utilized RNA sequencing and metabolic mass spectrometry to identify the molecular and metabolic feature of CARD10. The signalling pathway of CARD10 was verified by Western blotting analysis and functional assays. RNA sequencing and metabolic mass spectrometry of CARD10 knockdown identified the metabolic enzyme carbamoyl phosphate synthase 1 (CPS1) in the urea cycle as the downstream gene regulated by CARD10. We confirmed that CARD10 affected cell proliferation and nucleotide metabolism through regulating CPS1. We indicated that CARD10 promote bladder cancer growth via CPS1 and maybe a potential therapeutic target in bladder cancer.

## 1 INTRODUCTION

Bladder cancer is the most common type of urinary system cancer in the United States, with 80 470 new cases and 17 670 new deaths anticipated in 2019.^1^ Bladder cancer is usually diagnosed as the non‐muscle‐invasive bladder cancer (NMIBC) form. Approximately 20%‐40% of patients diagnosed with NMIBC will experience progression and recurrence.^2^

Caspase recruitment domain family member 10 (CARD10), also named CARD‐containing MAGUK protein 3 (CARMA3), is a member of the caspase activation and recruitment domain (CARD) family. Caspase recruitment domain family member 10 mainly assembles the CARMA‐BCL10‐MALT1 (CBM) signalosome, binding the hydrophilic region of the BCL10 and MALT1 proteins. The complex mainly responds to G protein‐coupled receptors (GPCRs) or growth factor receptor tyrosine kinases (RTKs). Then, CARD10 is phosphorylated and activates nuclear factor kappa B (NF‐κB) via the canonical IKK complex‐associated pathway, which phosphorylates IKK complex and IκBα to generate and translocate p50 (also named NF‐κB1) and p65 (also named RELA) into nucleus.^3^, ^4^ In addition, several microRNAs can regulate CARD10 via sponging.^5^, ^6^, ^7^

Caspase recruitment domain family member 10 expression is widely distributed in tissues, and overexpression has been reported in many solid tumours. In colon cancer, approximately 31% of samples exhibited overexpression.^8^ In non‐small‐cell lung cancer, approximately 70% of patients exhibited high CARD10 expression due to EGFR mutation.^9^ In breast cancer, 42% of cancer samples expressed a high level of CARD10, which was correlated with TNM stage,^10^ and in renal cell carcinoma, CARD10 expression was associated with tumour stage, size and metastasis.^11^ In our previous reports, we also found that 38.8% of bladder cancer tissue samples highly expressed CARD10.^12^

In the present study, we focused on the role of CARD10 in bladder cancer and analysed its downstream effect. We identified carbamoyl phosphate synthase 1 (CPS1), a metabolic enzyme that utilizes ammonia to produce carbamoyl phosphate, as a potential downstream gene of CARD10. In addition, we found that CARD10 impacted nucleotide metabolism through CPS1 in bladder cancer cells.

## 2 MATERIALS AND METHODS

### 2.1 Cell culture reagents

The human bladder cancer cell lines UMUC3 and T24 and the normal bladder epithelial cell line SVHUC1 were purchased from the Chinese Academy of Sciences Cell Bank and cultured in RPMI 1640 medium (Gibco) containing 10% foetal bovine serum (Gibco) at 37°C in an atmosphere of 5% CO_2_. Puromycin and hygromycin were purchased from Sigma, pyrrolidinedithiocarbamic acid (PDTC), l‐arginine and dNTPs were purchased from Beyotime, and tumour necrosis factor‐α (TNF‐α) was purchased from Apexbio.

### 2.2 Tissue samples

Tumour and adjacent tissue samples from 30 patients diagnosed with bladder cancer at the First Affiliated Hospital of China Medical University were collected and stored at −80°C. The tissue samples were collected and used in accordance with approval by the Institutional Ethics Committee of China Medical University. Informed consent forms were signed before surgery.

### 2.3 Reverse transcription quantitative polymerase chain reaction (RT‐qPCR)

Extraction of total RNA, synthesis of cDNA and PCRs were performed as previously described.^13^ The primer sequences are listed as follows: CARD10 (forward, 5′‐CCCCTAAGAGATCCTTCAGCAG‐3′; reverse, 5′‐CCACACGCTGTCAGAGGATG‐3′), CPS1 (forward, 5′‐ACTTCAGTTGAGTCCATTATGGC‐3′; reverse, 5′‐GGAACGGATCATCACTGGGTAG‐3′), GAPDH (forward, 5′‐GGAGCGAGATCCCTCCAAAAT‐3′; reverse, 5′‐GGCTGTTGTCATACTTCTCATGG‐3′), ASL (forward, 5′‐ATCACTCTCAACAGCATGGAT‐3′; reverse, 5′‐TGAATTCCTTGGTGCAGTAGAG‐3′), ASS1 (forward, 5′‐CTTCATGTACCTGAACGAAGTC‐3′; reverse, 5′‐TCGATGTCTAAATGAGCATGGT‐3′), OTC (forward, 5′‐CCGTGACCTTCTCACTCTAAAA‐3′; reverse, 5′‐AGCCTGTTTCTGTAGACAATCT‐3′), SLC25A13 (forward, 5′‐GAAATTTGGTCAGGTTACACCC‐3′; reverse, 5′‐ACTTGTAGAAGAACTGGTCGAG‐3′), and SLC25A15 (forward, 5′‐CAGTGTGGTCTGTCATCAAAAG‐3′; reverse, 5′‐TCGAAGTAAAGTGCTTGAGAGT‐3′).

### 2.4 Transient transfections

Cell transfection was performed by Lipofectamine 3000 (Life Technologies Corporation) and prepared for analysis 48 hours after transfection. Caspase recruitment domain family member 10, CPS1 and negative control (NC) siRNA (JTSBIO Co.) were designed as follows (5′‐3′): CARD10 (#1: forward, 5′‐GGCUCAAGGAUGAGAACUATT‐3′ and reverse, 5′‐UAGUUCUCAUCCUUGAGCCTT‐3′; #2: forward, 5′‐GAACCUUGCUCUGGAAUCATT‐3′ and reverse, 5′‐UGAUUCCAGAGCAAGGUUCTT‐3′), CPS1 CARD10 (#1: forward, 5′‐GCAUUGACCUAGUGAUUAATT‐3′ and reverse, 5′‐UUAAUCACUAGGUCAAUGCTT‐3′; #2: forward, 5′‐GCCCUUCAUCCUACCUCAATT‐3′ and reverse, 5′‐UUGAGGUAGGAUGAAGGGCTT‐3′).

### 2.5 Stable transfections

UMUC3 and T24 knockdown cells were constructed by shRNA (OBIO), using the same sequence as siCARD#1. Puromycin (10 µg/mL) was applied for selection, and GFP and Western blotting were used for confirmation. Stable CARD10 and CPS1 overexpression were established by plasmid transfection (JTSBIO Co.; Genechem). Plasmids were transfected with Lipofectamine 3000, and transfectants were selected with hygromycin (200 µg/mL) or puromycin (10 µg/mL). Cells were confirmed by RT‐qPCR and Western blotting.

### 2.6 Western blot analysis

Total protein was extracted from tissues and cell lines by lysis buffer (Beyotime). Nuclear proteins were extracted in accordance with the protocol of a Nuclear Extraction Kit (Abcam). Electrophoresis, transfer, antibody incubation and chemiluminescent detection were performed. The following primary antibodies were used: anti‐CARD10 (Abcam), anti‐phospho‐IκBa (Ser32/36) (CST), anti‐CPS1 (Proteintech), anti‐NF‐κB1 (CST), anti‐histone H3 (CST), and anti‐GAPDH.

### 2.7 Cell proliferation assay

Treated cells were seeded in 96‐well plates, Cell Counting Kit‐8 (CCK‐8) assay reagent (Dojindo Molecular Technologies) was added, and the absorbance at 450 nm was measured by an absorbance reader (Bio‐Rad).

### 2.8 Flow cytometry assay

Treated cells were used for an apoptosis assay with a FITC Annexin V Apoptosis Detection Kit (BD Pharmingen) and a cell cycle assay with PI/RNase Staining Buffer Solution (BD Pharmingen), or a Cell‐Light 5‐ethynyl‐2′‐deoxyuridine (EdU) DNA Cell Proliferation Kit (Beyotime) following the manufacturers' protocols and were analysed by flow cytometry (Becton Dickinson Biosciences) as previously described.^14^

### 2.9 ChIP‐PCR assay

A total of 10^7^ cells were prepared, and the ChIP‐PCR assay was performed as described in the manufacturer's protocol for the SimpleChIP Plus Sonication Chromatin IP Kit (CST). The promoter region was predicted with jaspar.genereg.net, and primers were designed according to the highest‐scoring region (forward, 5′‐AGAGGTGAGATCAAGGCGTAAAC‐3′; reverse, 5′‐CCCAACCTAGAGAACTGAGGACT‐3′).

### 2.10 Ammonia assay and urea assay

According to other studies, CPS1 not only participates in urea metabolism but also affects pyrimidine synthesis.^15^, ^16^ As ammonia was utilized by CPS1 to produce carbamoyl phosphate, the inhibition of CPS1 expression may increase the ammonia level.^17^ Cells were transfected with siRNA and treated as described in the ammonia assay kit (Sigma) and QuantiChrom Urea Assay Kit (BioAssay Systems) protocols. The absorbance at 450 nm was measured by an absorbance reader (Bio‐Rad).

### 2.11 Metabolic mass spectrometry (MS) and RNA sequencing

mRNA was sequenced using an Illumina HiSeq™ 2500, and metabolites were analysed by MS on a Q Exactive Orbitrap mass spectrometer (Thermo Fisher Scientific). RNA sequencing was performed in three biological replicates of CARD10 knockdown UMUC3 cells. Metabolomics analysis in both positive and negative ion modes was performed in CARD10 knockdown UMUC3 cells with six biological replicates, and the data were classified by a multivariate statistical analysis model, orthogonal partial least squares discriminant analysis (OPLS‐DA), and verified by permutation tests. Pearson correlation coefficient was also calculated between CARD10 and other genes or metabolites in the two omics data.

### 2.12 In vivo analysis

Xenograft experiments in nude mice (BALB/C nude, female, 4 weeks) were performed at the experimental animal centre of China Medical University. Ten mice were randomized into two groups, and a total of 1.5 × 10^7^ control and stably transfected UMUC3 cells were subcutaneously injected. All mice were housed, and tumours were harvested after 5 weeks of observation. Tumour volume was estimated by the following formula: volume = 0.5 × length × width^2^. The experiments were conducted in accordance with institutional guidelines and approved by the Animal Care and Use Committee.

### 2.13 Immunohistochemical (IHC) analysis

Paraffin‐embedded tissues from nude mice were cut into 4‐μm slices. Immunohistochemical analysis was performed as previously described.^14^

### 2.14 Statistical analysis

Experimental data are presented as the means ± SDs of at least three independent experiments performed with experimental triplicates. Differences between groups were analysed by Student's *t* test or two‐way ANOVA. Wilcoxon test was used for significant analysis for clinical samples. *P* values of < 0.05 were considered statistically significant (* represents *P* < .05, ** represents *P* < .01 and *** represents *P* < .001). Data analyses were carried out using GraphPad Prism 7.0 (GraphPad Software).

## 3 RESULTS

### 3.1 Knockdown of CARD10 down‐regulated CPS1 and inhibited tumour growth

**Figure 1:**
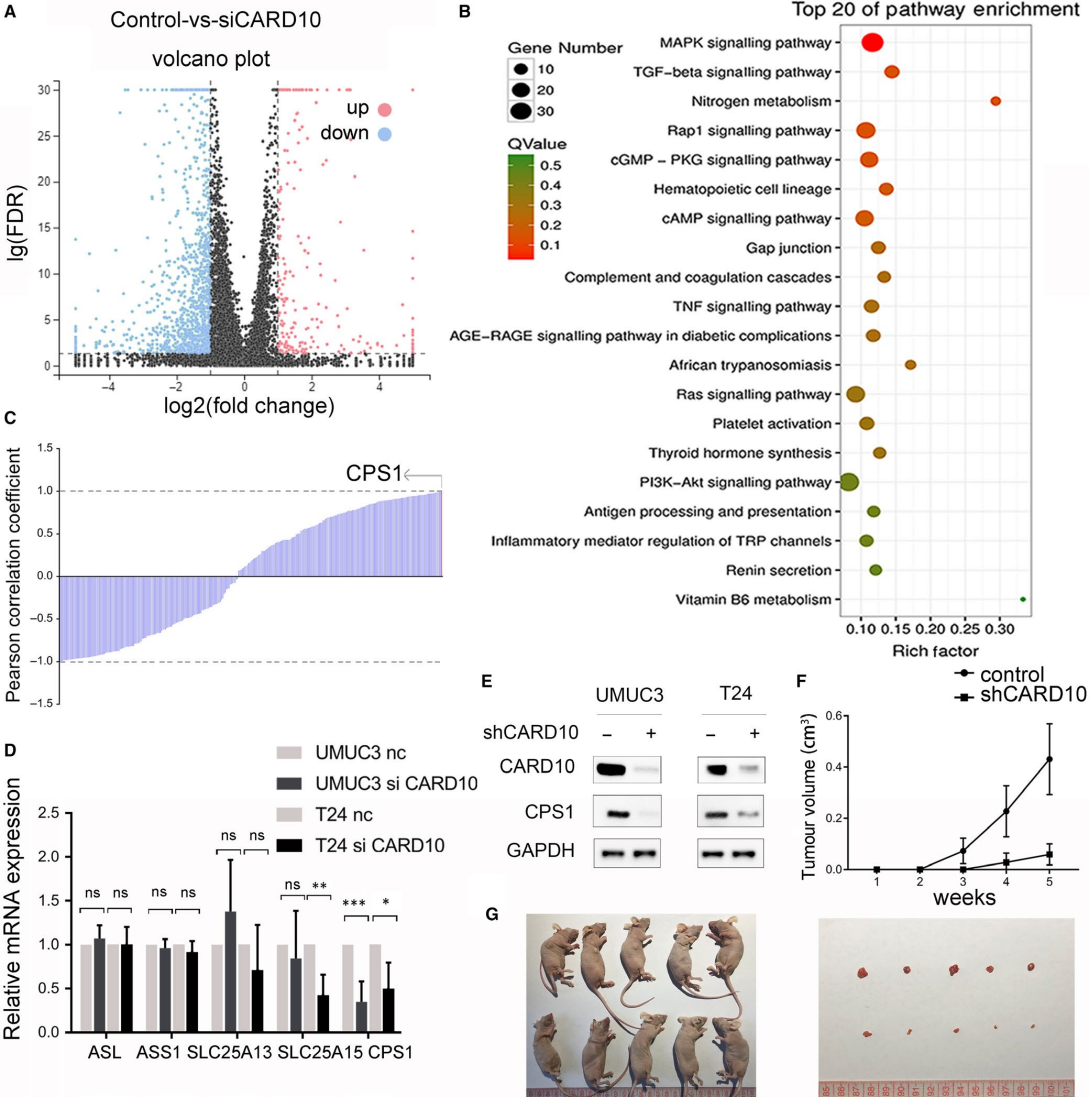
Knockdown of CARD10 down‐regulated CPS1 and inhibited tumour growth. A, The different expressed genes (DEGs) in RNA sequencing showed in volcano plot. B, KEGG pathway database analysis of DEGs, pathways were ranked by *P* value. C, The Pearson correlation coefficient of expression levels between CARD10 and genes in MAPK, TGF‐beta and nitrogen pathway, CPS1 expression showed the strongest association with CARD10 expression. D, The expression level of genes in urea metabolism, measured by RT‐qPCR. E, The protein expression level of CPS1 in shCARD10 cell lines. F, Left: growth curve of tumour volume and images of xenograft nude mice. Right: the IHC analysis of xenograft tumour

We knocked down CARD10 expression with two different siRNAs (Figure S1) and performed RNA sequencing to explore the downstream molecular mechanism. A total of 212 up‐regulated genes and 1103 down‐regulated genes with a cut‐off value of log2 (fold change) >1 and a *t* test *P* value of <.01 (Figure 1A) were filtered out. Enrichment of the differentially expressed genes was investigated by KEGG pathway database analysis and ranked by *P* value (Figure 1B). The Pearson correlation coefficient was calculated for genes in the top three enriched pathways, and CPS1 expression showed the strongest association with CARD10 expression (Figure 1C). To further confirm this result, we performed RT‐qPCR to evaluate the changes in the expression of several genes related to CPS1 (Figure 1D), including ASL, ASS1, CPS1, SLC25A13 and SLC25A15, only CPS1 was significantly down‐regulated. The regulation of CPS1 expression by CARD10 was further investigated with the stable CARD10 knockdown cell line (Figure 1E). The xenograft model showed that decreased expression of CARD10 inhibited tumour growth and down‐regulated CPS1 expression in vivo (Figure 1F).

### 3.2 CARD10 regulated the expression of CPS1 via the NF‐κB pathway

**Figure 2:**
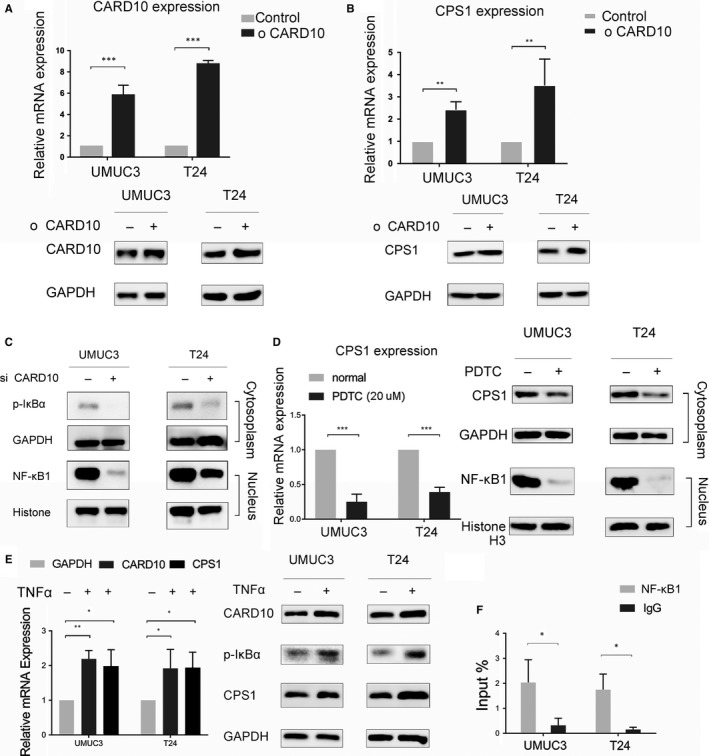
CARD10 regulated the expression of CPS1 via the NF‐κB pathway. A, RNA and protein expression levels of CARD10 in control and CARD10 overexpressed cell lines. B, RNA and protein expression levels of CPS1 in control and CARD10 overexpressed cell lines. C, The phosphorylation level of IκBα in the cytoplasm and the expression level of NF‐κB1 in the nucleus interfered by siCARD10. D, RNA and protein expression levels of CPS1 when treated with NF‐κB inhibitor PDTC in 20 μM. E, RNA and protein expression levels of CPS1 when treated by NF‐κB activator TNFα in 6 μg/ml. F, DNA amplification of CPS1 promoter region in CHIP‐PCR assays precipitated by NF‐κB1 or IgG antibody compared to input DNA

CARD10‐overexpressing cell lines were established (Figure 2A), and an elevation in the CPS1 level was observed (Figure 2B). Next, we sought to determine whether CPS1 was regulated by CARD10 via the downstream NF‐κB signalling pathway. The phosphorylation level of IκBα in the cytoplasm and the expression of NF‐κB1 in the nucleus decreased when CARD10 was knocked down (Figure 2C). An NF‐κB activation inhibitor, PDTC, was used at 20 μM in bladder tumour cell lines, and the blockade of NF‐κB activation significantly decreased CPS1 expression (Figure 2D). Another NF‐κB pathway agonist, TNF‐α, slightly increased the expression of both CARD10 and CPS1 (Figure 2E). Primers were designed according to the promoter regions predicted for binding between the transcription factor NF‐κB1 and CPS1. A ChIP‐PCR assay was performed, and a slight increase compared with IgG precipitation was shown for the first primer, indicating a potential binding region of NF‐κB1 to promote CPS1 transcription (Figure 2F).

### 3.3 CPS1, as a downstream gene of CARD10, inhibited cell proliferation and increased apoptosis

**Figure 3:**
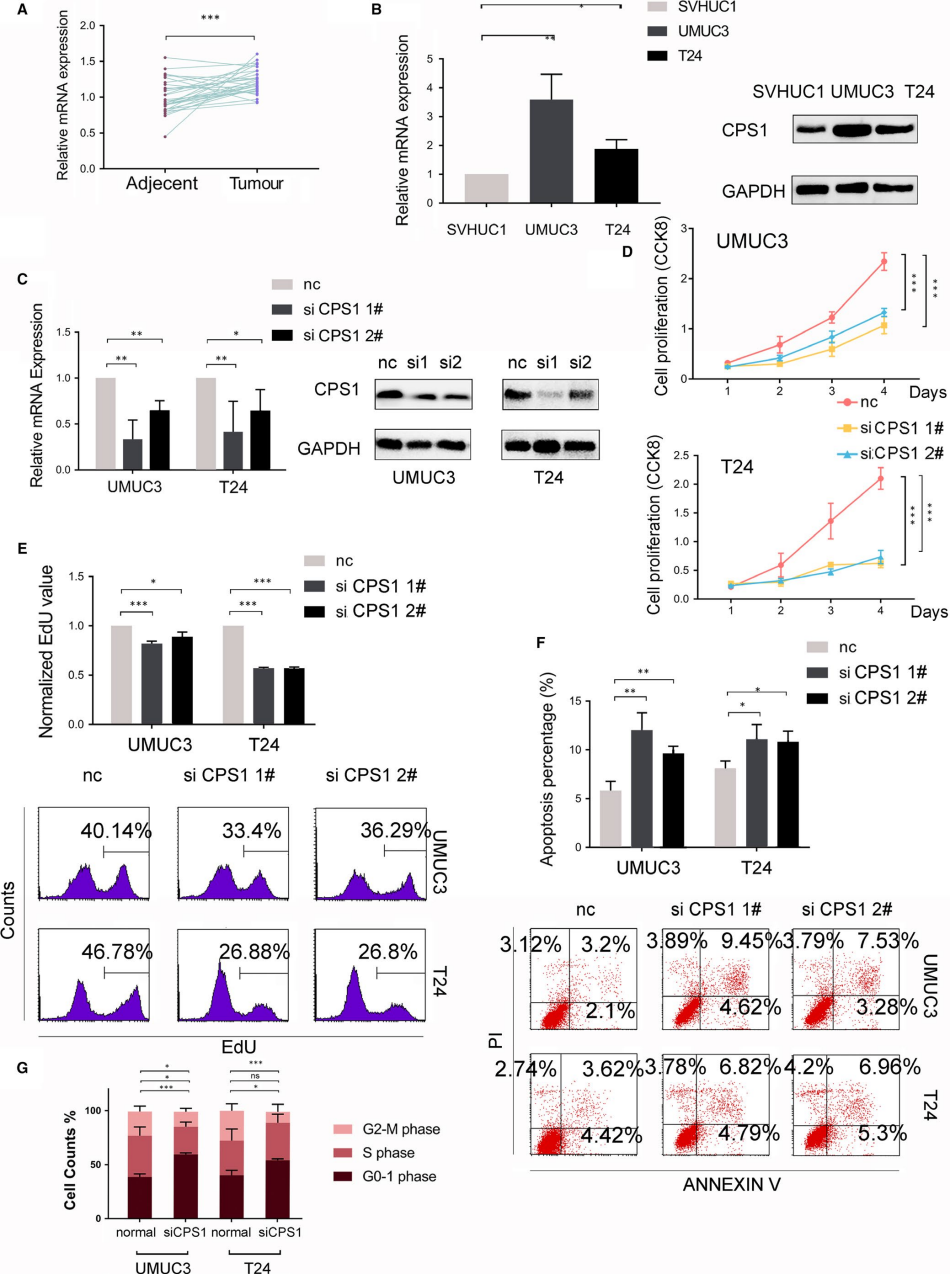
CPS1, as a downstream gene of CARD10, inhibited cell proliferation and increased apoptosis. A, CPS1 expression level of 30 pairs of bladder tumour samples by RT‐qPCR. B, Effect of CPS1 expression on BLCA patients' survival from TCGA database. C, CPS1 expression levels of SVHUC1, UMUC3 and T24 cell lines. D, RNA and protein expression levels of CPS1 knocked down by two different siRNAs. E, Cell proliferation of UMUC3 and T24 measured by CCK‐8 assays. F, Cell proliferation of UMUC3 and T24 measured by EdU and analysed by flow cytometry. G, The apoptosis analysis of UMUC3 and T24 cells treated by siCPS1s. H, The cell cycle analysis of UMUC3 and T24 by siCPS1

Carbamoyl phosphate synthase 1 expression in tissue was assessed in the 30 pairs of bladder tissue samples (Figure 3A), and all samples were selected from urothelial carcinoma patients according to their pathological results (Table S1). Survival data from the TCGA database showed that high expression of CPS1 was related to a worse prognosis in patients with bladder cancer (Figure 3B). We further found that the expression of CPS1 was higher in the bladder cancer cell lines UMUC3 and T24 than in the SVHUC1 cell line (Figure 3C).

The CPS1 gene was knocked down using two different siRNAs (Figure 3D). A CCK‐8 assay was used to measure cell proliferation (Figure 3E). Additionally, an EdU assay with flow cytometric analysis was used to determine the DNA synthesis rate to indicate cell proliferation. The results showed a significant decrease in the CPS1 knockdown groups compared with the negative control group (Figure 3F). In addition, the apoptosis assay revealed an increase in apoptotic cells (Figure 3G). These results showed that similar to CARD10 knockdown, CPS1 knockdown affected cell apoptosis and cell proliferation. Considering that CPS1 participates in nucleotide synthesis, we hypothesized that it might function in the cell cycle. Flow cytometric analysis showed that CPS1 knockdown arrested cells in G0/G1 phase and reduced the percentage of cells in the S and G2/M phases (Figure 3H).

### 3.4 CPS1 overexpression restored the proliferation‐inhibitory effect of CARD10 knockdown

**Figure 4:**
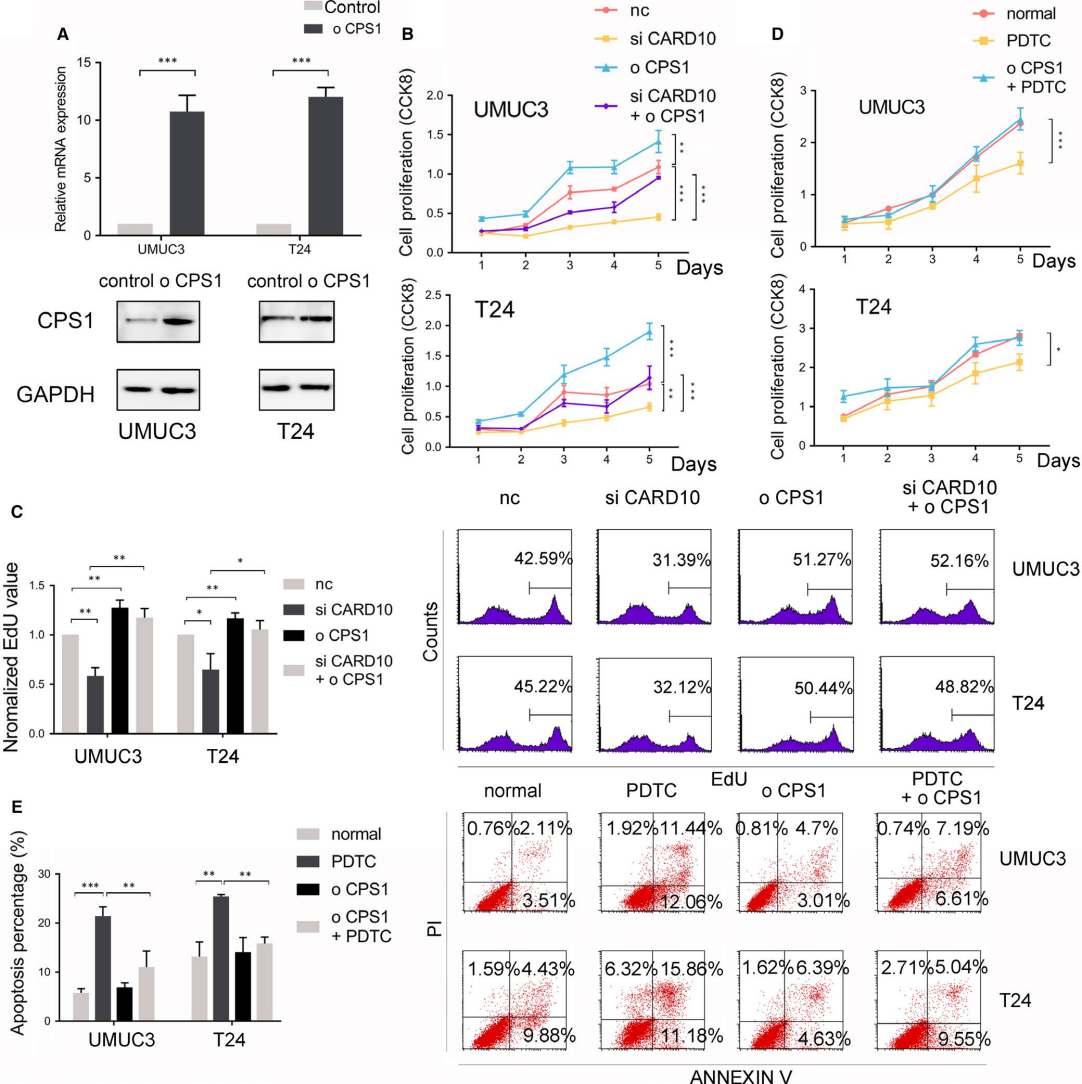
CPS1 overexpression restored the proliferation‐inhibitory effect of CARD10 knockdown. A, RNA and protein expression levels of CPS1 overexpression. B, Cell proliferation of UMUC3 and T24, treated with siCARD10 and CPS1 overexpression, measured by CCK‐8. C, Cell proliferation of UMUC3 and T24, treated with siCARD10 and overexpression of CPS1, measured by EdU. D, CCK‐8 assays of PDTC treatment in normal and overexpressed CPS1 cell lines. E, The apoptosis analysis of PDTC treatment in normal and CPS1 overexpression cell lines

CPS1‐overexpressing cell lines were established (Figure 4A), and CCK‐8 and EdU assays were performed. Caspase recruitment domain family member 10 knockdown inhibited but CPS1 overexpression promoted cell proliferation, and CPS1 overexpression in CARD10 knockdown cells markedly increased the cell proliferation rate (Figure 4B,C). Next, we used PDTC to mimic CARD10 knockdown because the NF‐KB pathway might act as an intermediate element in regulating downstream gene expression. In this assay, CARD10 knockdown cells showed patterns similar to those described above for cell proliferation (Figure 4D). Nuclear factor kappa B pathway was critical in protecting cells from apoptosis, and the use of its inhibitor, PDTC, significantly induced this programmed cell death. As the potential downstream protein of NF‐KB pathway, overexpression of CPS1 significantly protected against the apoptosis effect induced by PDTC (Figure 4E).

### 3.5 Metabolomic analysis indicated that CARD10 affects nitrogen metabolism by regulating CPS1

**Figure 5:**
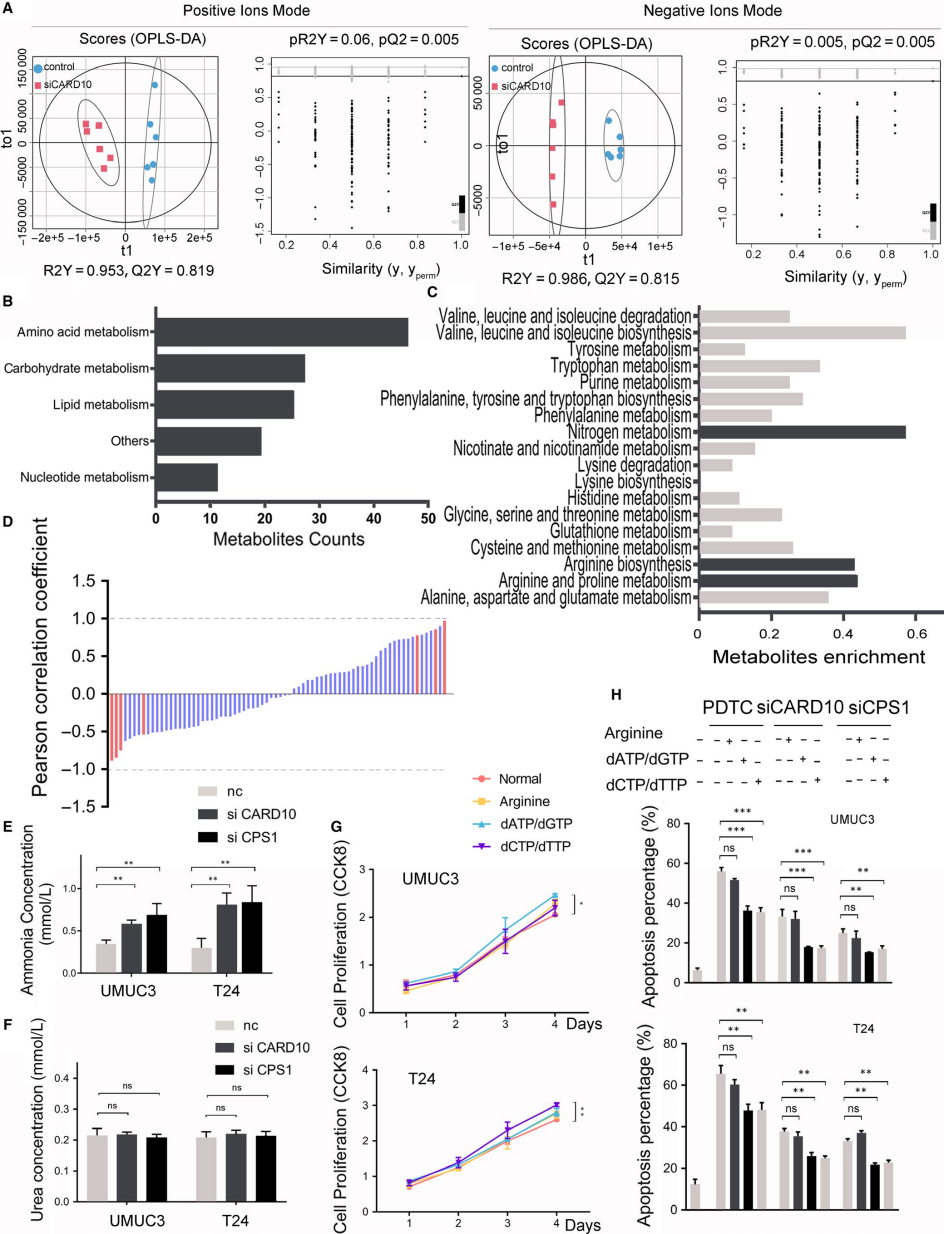
Metabolic effect of CARD10 and CPS1. A, The OPLS‐DA and permutation test of cells divided into siCARD10 and negative control groups. B, Metabolites sorted by OPLS‐DA were annotated and classified by KEGG compound database. C, The KEGG enrichment analysis of metabolites related in aminoacid metabolism pathways. D, The Pearson correlation coefficient between CARD10 and aminoacid metabolism‐related metabolites in the omics data. The red column represents metabolites involved in nitrogen metabolism. E, Ammonia level of siCARD10 treated cell lines. F, Urea expression level of cell lines treated with siCARD10 or siCPS1. G, CCK‐8 assays of arginine, purine or pyrimidine supplement (100 μM) in UMUC3 and T24. H, The apoptosis analysis of arginine, purines or pyrimidines supplied in UMUC3 and T24, treated with siCARD10 or siCPS1

Caspase recruitment domain family member 10 knockdown caused a significant change in the metabolites in bladder cancer cells (Figure 5A). Among the metabolites detected by MS, 339 compounds were matched with KEGG compound annotations. A total of 114 metabolites were sorted by a threshold defined by a VIP score of >1 and a *t* test *P* value of <.05 for further analysis. KEGG enrichment analysis indicated that 46 of these metabolites were associated with aminoacid metabolism (Figure 5B) and were mainly enriched in urea cycle‐related pathways, such as nitrogen metabolism, arginine and proline metabolism and arginine biosynthesis (Figure 5C). Furthermore, we found that the metabolites involved in nitrogen metabolism were more sensitive to variations associated with CARD10 expression among aminoacid‐related metabolism pathways (Figure 5D).

### 3.6 The metabolic effect of CARD10 and CPS1

The accumulation of ammonia showed that knocking down CARD10 might have a similar effect as the inhibition of CPS1 expression (Figure 5E). The level of urea was measured to estimate the integral change in the urea cycle, and the results showed that neither CARD10 nor CPS1 knockdown affected the urea level (Figure 5F). Arginine, purines or pyrimidines, products of either urea metabolism or nucleotide synthesis, which CPS1 participates in, were added, and their function in proliferation was evaluated by a CCK‐8 assay. The results indicated that pyrimidines might slightly promote cell growth (Figure 5G). According to these metabolic effects, we supplied arginine and different nucleotides to cells treated with siRNAs or PDTC. Apoptosis was reduced when cells were restored by these metabolites (Figure 5H).

## 4 DISCUSSION

In our previous reports, we found that CARD10 was highly expressed in bladder cancer tissue samples and cell lines. And knockdown of CARD10 suppressed bladder tumour cell (UMUC3 and T24) proliferation and promoted apoptosis, thus we supposed CARD10 might act as an oncogene in bladder cancer.^12^, ^18^ CARD10 plays a critical role in intracellular signalling transduction and impacts many oncologic processes in tumour proliferation, metastasis, angiogenesis, and stemness and chemotherapeutic resistance.^19^, ^20^, ^21^ These characteristics may render it a therapeutic target. In this study, we focused on the downstream effect of CARD10 on tumour growth in bladder cancer.

The main intrinsic factors that promote tumour growth usually contribute to abnormal expression of cell cycle‐related proteins or metabolic genes in bladder cancer.^22^, ^23^ Therefore, we performed metabolic MS and RNA sequencing to identify the metabolic and molecular features of CARD10. The metabolomics data indicated that CARD10 mainly affects urea metabolism, especially nitrogen metabolism. In addition, the RNA sequencing data revealed a gene involved in nitrogen metabolism, CPS1, as a potential downstream gene regulated by CARD10. Additionally, the RNA sequencing data revealed several other genes that have been reported to be regulated by the NF‐κB pathway, such as IL6, MAPK1, SSAT, HK2 and GLUT1.^24^, ^25^, ^26^, ^27^, ^28^ Thus, we hypothesized that CPS1 may also be regulated via the NF‐κB pathway.

Carbamoyl phosphate synthase 1 is a core gene in urea metabolism and nucleotide synthesis, which mainly occur in mitochondria. Abnormal and ectopic CPS1 expression in tumour cells may lead to urea cycle deficiency and a nucleic acid pool imbalance.^15^, ^16^, ^29^ CPS1 overexpression is associated with poor prognosis in several types of cancer, such as colon cancer, cholangiocarcinoma and glioblastoma.^30^, ^31^, ^32^ CPS1 has also been reported to be regulated by hepatocyte nuclear factor 3β (HNF3β) and sirtuin 5 (SIRT5) by their transcriptional activation or deacetylation‐ and deglutarylation‐mediated activation.^33^, ^34^, ^35^ Thus, the results of our study might supplement the previous reported associations.

Regarding the metabolic effect, we concluded that CARD10 overexpression mostly affected nucleotide metabolism rather than aminoacid metabolism due to ectopic expression of CPS1. Cytosolic CPS1 may function as its homologous protein carbamoyl phosphate synthetase 2, aspartate transcarbamylase and dihydroorotase (CAD) in nucleotide synthesis.^29^ In addition, CPS1 and nucleotide supplementation exhibited a significant protective effect on apoptosis induced by silencing CARD10 or NF‐κB. We hypothesized CPS1 activation might affect the process of chemotherapy‐induced DNA damage similar to CARD10 and NF‐κB.^21^

As a research study, cell models used in our study lacked the relevance to a real‐life situation, which might cause a shifting effect from clinical bladder cancer, especially in metabolic effects. Although our study utilized several methods to confirm the relationship between CARD10 and CPS1, it still has limitations. First, whether other transcription factors could affect CPS1 transcription was not investigated. Next, an accurate evaluation of nucleic acids under interference with CARD10 and CPS1 expression might be warranted. In addition, further experiments to clarify the genetic mechanism of CPS1 might provide more rigorous support for our findings. Therefore, additional studies are essential in order to understand the active molecular mechanism in bladder cancer.

## CONFLICT OF INTEREST

All authors give consent for the publication of the manuscript. The authors declare no competing financial interests. All authors read and approved the final manuscript.

## AUTHOR CONTRIBUTIONS

Zhe Zhang and Chuize Kong designed the research. Xi Liu performed the experiments, analysed the data and prepared the manuscript. Xiaotong Zhang provided material support. Jianbin Bi and Zhenhua Li supervised on experimental processes and edited the manuscript.

## Data Availability

The data sets used and analysed during the current study are available from the corresponding author upon reasonable request.
