# Supplementary figures and images for: Caspase recruitment domain family member 10 regulates carbamoyl phosphate synthase 1 and promotes cancer growth in bladder cancer cells
Source: J Cell Mol Med. 2019 Sep 29;23(12):8128–38. doi: 10.1111/jcmm.14683 (PMC6850932; doi:10.1111/jcmm.14683)

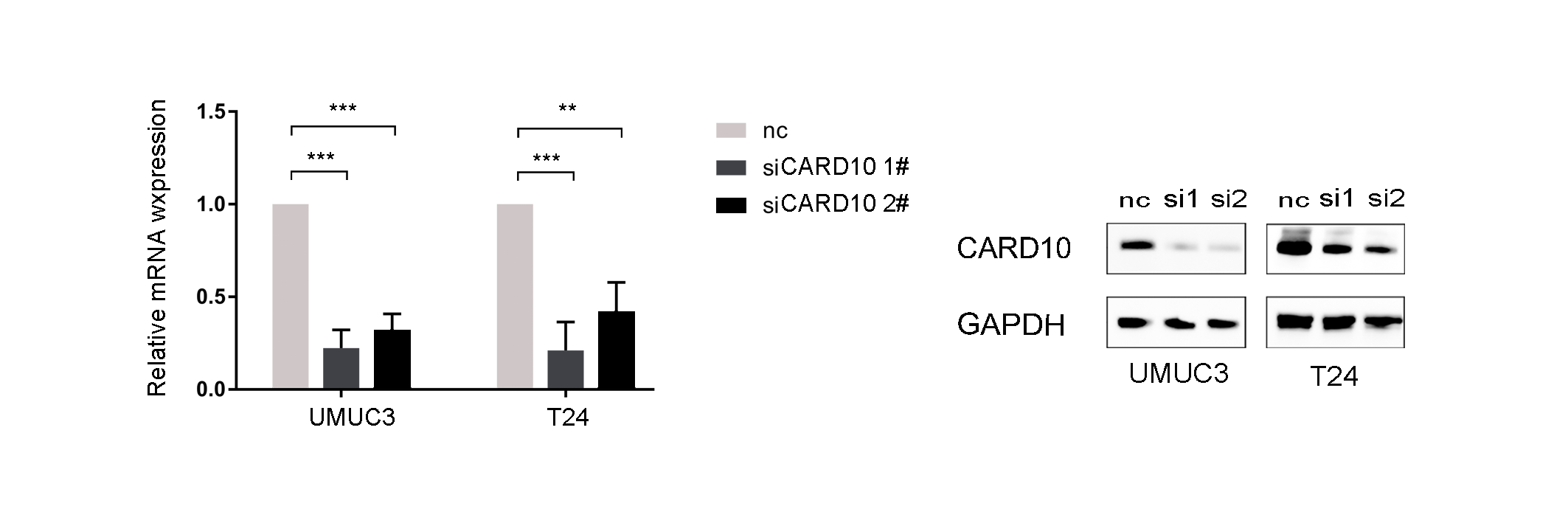

Supplement: Supplementary file 1 [file JCMM-23-8128-s001.tif]
