# Supplementary material for: Caspase recruitment domain family member 10 regulates carbamoyl phosphate synthase 1 and promotes cancer growth in bladder cancer cells
Source: J Cell Mol Med. 2019 Sep 29;23(12):8128–38. doi: 10.1111/jcmm.14683 (PMC6850932; doi:10.1111/jcmm.14683)
Supplement: Supplementary file 2 [file JCMM-23-8128-s002.docx]

| Table S1 patient features of 30 urothelial carcinoma patients | |
| --- | --- |
| Characteristics | Case |
| All cases | 30 |
| Age(years) |  |
| <60 | 11 |
| ≥60 | 19 |
| Gender |  |
| Male | 25 |
| Female | 5 |
| TNM stage |  |
| pTa-pT1 | 10 |
| pT2-pT4 | 20 |
| Histological grade |  |
| Low grade | 4 |
| High grade | 26 |
| Tumor type |  |
| primary | 28 |
| recurrence | 2 |
